# Safety of the neprilysin/renin-angiotensin system inhibitor LCZ696

**DOI:** 10.18632/oncotarget.18312

**Published:** 2017-05-31

**Authors:** Bo Li, Yunhe Zhao, Bo Yin, Mengfei Helian, Xinmei Wang, Feng Chen, Hongxia Zhang, Hui Sun, Bin Meng, Fengshuang An

**Affiliations:** ^1^ Department of Cardiology, Central Hospital of Zibo, Zibo, 255036, PR China; ^2^ Department of Pathology, Central Hospital of Zibo, Zibo, 255036, PR China; ^3^ Department of Cardiology, Qilu Hospital of Shandong University, Ji’nan, 250012, PR China

**Keywords:** heart failure, hypertension, LCZ696, sacubitril/valsartan, safety

## Abstract

**Objectives:**

The combined neprilysin/rennin-angiotensin system inhibitor sacubitril/valsartan (LCZ696) has shown its superiority over ACEI/ARB therapy. In view of the existing concern of its adverse effects, we aimed to provide evidence of the safety of the new drug.

**Results:**

A total of 6 randomized trials with 11,821 subjects were included in this analysis. No significant differences were found in any adverse effects between LCZ696 and ACEI/ARB or placebo groups. LCZ696 significantly decreased the risks of serious adverse events and death compared with ACEI/ARB. LCZ696 also significantly decrease the risk of discontinuation of treatment for any adverse event no matter compared with ACEI/ARB or a placebo. LCZ696 significantly increased the risk of angioedema and dizziness, while it decreased the risk of renal dysfunction and bronchitis. There was no difference for hypotension, hyperkalemia, cough, upper respiratory tract inflammation, diarrhoea, back pain, nasopharyngitis, headache and influenza between the LCZ696 group and the ACEI/ARB group.

**Materials and Methods:**

A meta-analysis of eligible studies that used LCZ696 in heart failure and hypertension was performed. Embase, PubMed and the Cochrane Library were searched for randomized controlled trials (RCTs) with data on any adverse effects, serious adverse events, discontinuation of treatment for any adverse event, death, angioedema, hypotension, hyperkalemia, and other adverse effects to perform this meta-analysis.

**Conclusions:**

In addition to the beneficial effect of LCZ696 on end point events, the available evidences showed that LCZ696 was associated with less drug-risks than a placebo and ACEI/ARB.

## INTRODUCTION

Neprilysin, a neutral endopeptidase, has been seen as a potential therapeutic target in heart failure and hypertension because of its potent cardiorenal protective effects due to vasodilation, natriuresis, diuresis and attenuation of hypertrophy and fibrosis [1]. Inhibition of neprilysin can increase the level of NPs, and several drugs involved in inhibiting neprilysin were developed, such as ecadotril, racecadotril, and candoxatril. But these agents did not display efficient effects compared with traditional drugs [2–4]. The reason why neprilysin inhibition alone didn’t exert a significant effect is because of the promotive effect of neprilysin inhibition on plasma Ang II concentration and the restraining effect on metabolic clearance of Ang II in the medium [5]. Therefore, combined inhibition of both neprilysin and the renin–angiotensin system is seen as a plausible direction in the field. Omapatrilat, the first-in-class neprilysin and angiotensin-converting enzyme inhibitor, was developed. Although omapatrilat showed its promising potential, further development of omapatrilat was discontinued because of an increased incidence of angioedema [6].

LCZ696 (sacubitril/valsartan), which consists of the neprilysin inhibitor prodrug sacubitril (AHU377) and the ARB valsartan in a 1:1 molar ratio, provides simultaneous neprilysin inhibition and angiotensin-II receptor blockade. Different from omapatrilat, the design of LCZ696 mainly blocks ang-II, but not ACE, which lowers the risk of angioedema. And from the researches before, LCZ696 has shown greater blood pressure reduction in patients with hypertension [7, 8] and reduced all-cause mortality in heart failure patients compared with valsartan or enalapril [9, 10]. While results from individual clinical trials[7–10] and a meta-analysis [4] have confirmed the beneficial effects of LCZ696 and it was reported to be well tolerated in patients in individual clinical trials [11, 12], but the incidences of the side effects reported in each trial were still different. The purpose of this meta-analysis is to compare the safety of all published randomized controlled trials (RCTs) using LCZ696 inhibitors versus ACEI or ARB or a placebo for treating patients with heart failure and hypertension.

## RESULTS

### Any adverse events

**Figure 1:**
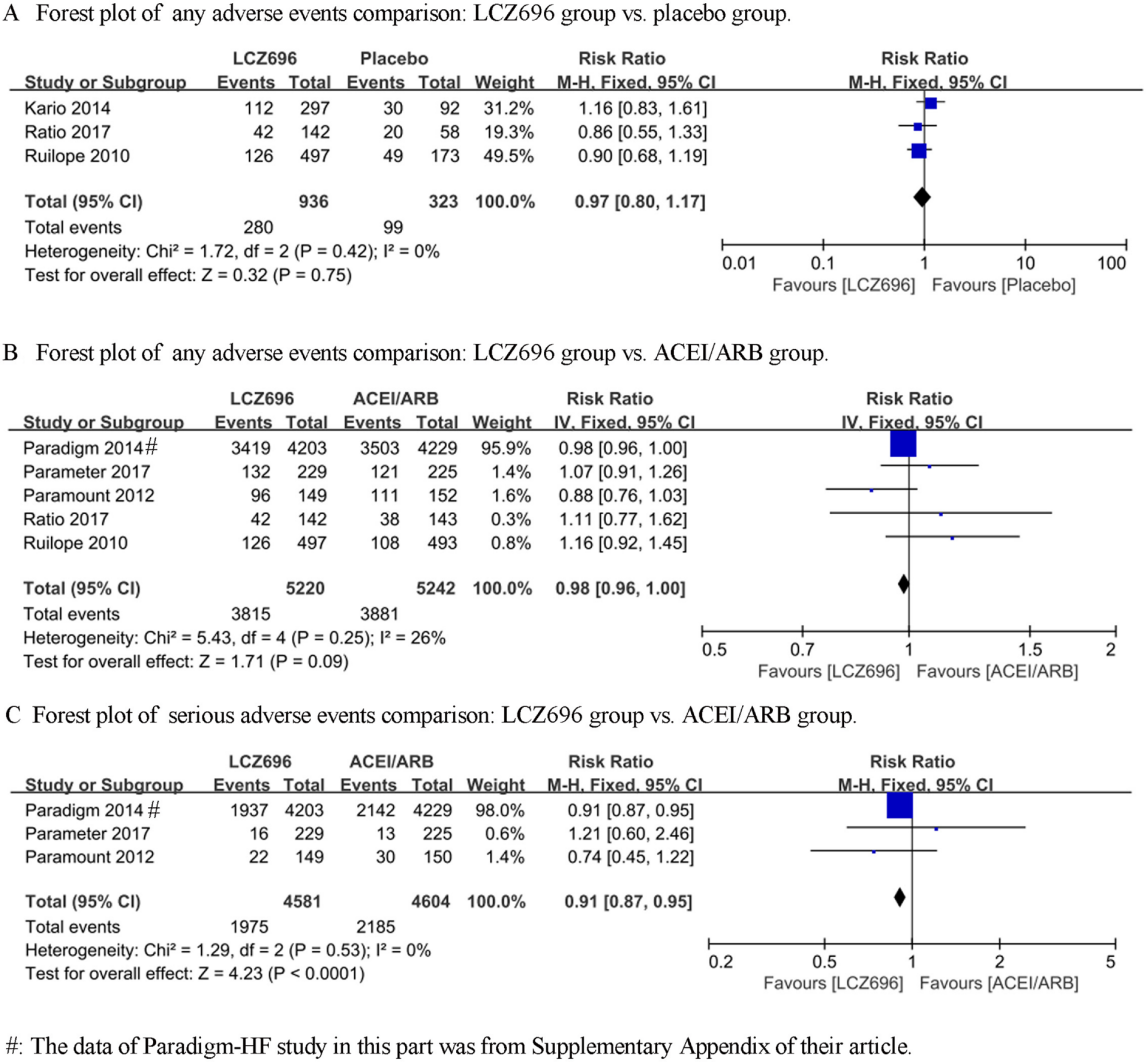
Forest plots depicting the comparison of LCZ696 and a placebo or ACEI/ARB on any adverse events, serious adverse events and discontinuation of treatment for any adverse event

We firstly compared the difference between LCZ696 and a placebo (Ruilope’s study [7], Kario’s study [8] and Ratio study [13] were included). The heterogeneity test result of these studies was calculated as I^2^ = 0%. Therefore, the fixed effect model was used for further analyses, and the results demonstrated that there was no difference between LCZ696 group and placebo group [RR = 0.97, 95% CI (0.80, 1.17), Z = 0.32, *P* = 0.75] (Figure 1A). Then, we compared the difference between LCZ696 and ACEI/ARB (Ruilope’s study [7], PARAMOUNT study [10], PARADIGM-HF study [9], Ratio study [13] and Parameter Study [14] were included). The heterogeneity test result of these studies was calculated as I^2^ = 26%. Therefore, the fixed effect model was used for further analyses, and the results demonstrated that there was no difference between the LCZ696 group and the ACEI/ARB group [RR = 0.98, 95% CI (0.96, 1.00), Z = 1.71, *P* = 0.09] (Figure 1B).

### Serious adverse events

For serious adverse events, we collected the data involving LCZ696 versus ACEI/ARB from the PARAMOUNT study, PARADIGM-HF study and Parameter Study. The heterogeneity test result of these studies was calculated as I^2^ = 0%. Therefore, the fixed effect model was used for further analyses, and LCZ696 showed a significant decrease in serious adverse events compared with the ACEI/ARB group [RR = 0.91, 95% CI (0.87, 0.95), Z = 4.23, *P* < 0.0001] (Figure 1C).

### Discontinuation of treatment for any adverse event

**Figure 2:**
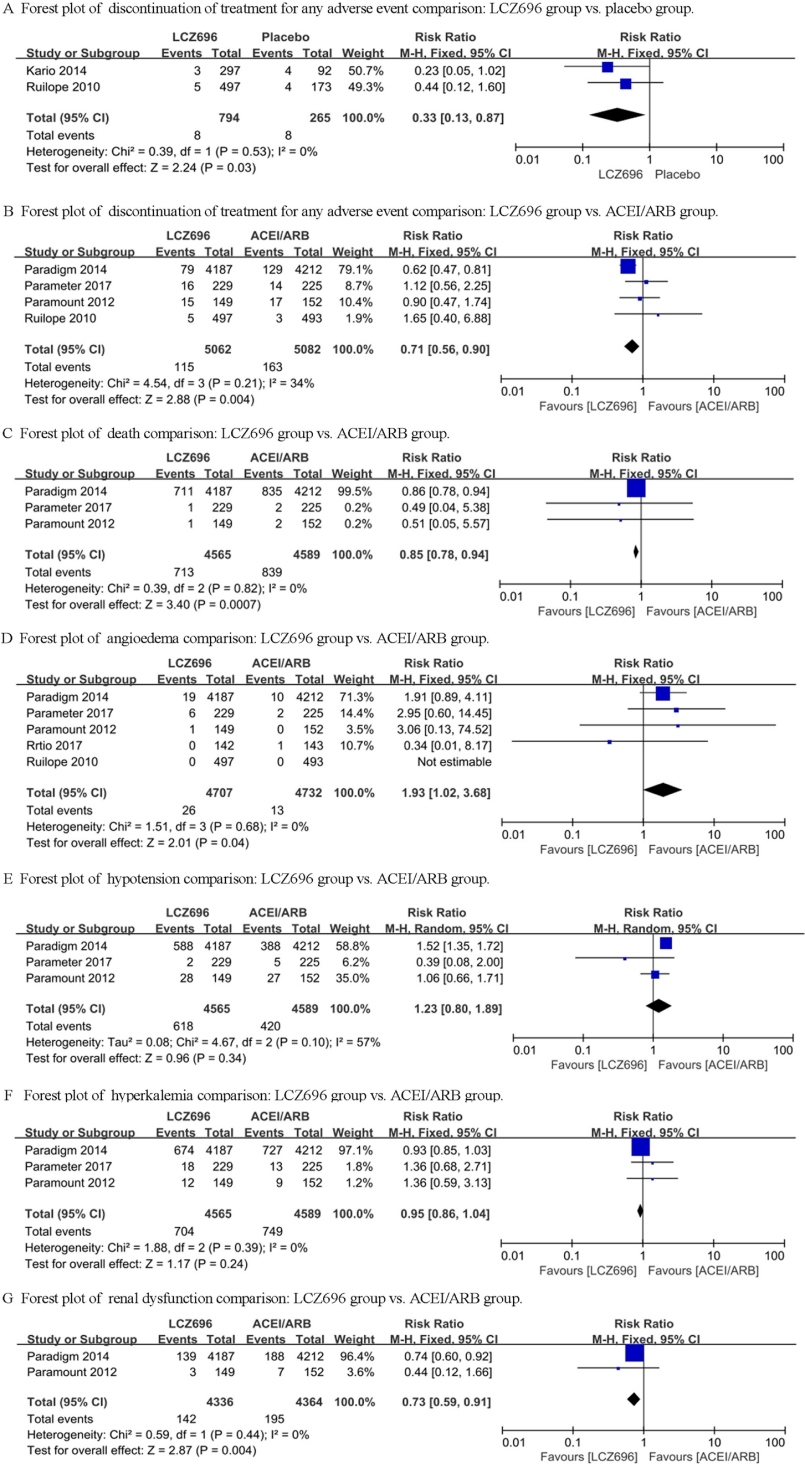
Forest plots depicting the comparison of LCZ696 and a placebo or ACEI/ARB on death, angioedema, hypotension, hyperkalemia and renal dysfunction

We first compared the difference between LCZ696 and a placebo. The heterogeneity test result of the 2 studies (Ruilope’s study and Kario’s study) was calculated as I^2^ = 0%. Therefore, the fixed effect model was used for further analyses, and LCZ696 showed a significant decrease in discontinuation of treatment for any adverse event compared with the placebo group [RR = 0.33, 95% CI (0.13, 0.87), Z = 2.24, *P* = 0.03] (Figure 2A). Then, we compared the difference of discontinuation of treatment for any adverse event between LCZ696 and ACEI/ARB. The heterogeneity test result of the 4 studies (Ruilope’s study, PARAMOUNT study, PARADIGM-HF study and Parameter Study) was calculated as I^2^ = 34%. Fixed effect model was used and LCZ696 showed a significant decrease in discontinuation of treatment for any adverse event compared with ACEI/ARB group [RR = 0.71, 95% CI (0.56, 0.90), Z = 2.88, *P* = 0.004] (Figure 2B).

### Death

In Ruilope’s study and Kario’s study, no case of death was reported. In PARAMOUNT study and Parameter study, 1 and 2 cases of death were reported respectively in the LCZ696 group and the valsartan/olmesartan group, while in PARADIGM-HF study, 711 and 835 cases of death were reported respectively in LCZ696 group and enalapril group. The heterogeneity test result of the 3 studies was calculated as I^2^ = 0%. The fixed effect model was used and the results demonstrated that the LCZ696 group significantly decreased death from any cause compared with the ACEI/ARB group [RR = 0.85, 95% CI (0.78, 0.94), Z = 3.40, *P* = 0.0007] (Figure 2C).

### Angioedema

From Ruilope’s study, the PARAMOUNT study, the PARADIGM-HF study, the Ratio study and the Parameter Study, we collected and analyzed the data of the difference between LCZ696 and ACEI/ARB. The heterogeneity test result of these studies was calculated as I^2^ = 0%. The fixed effect model was used and the results demonstrated that there was no difference between the LCZ696 group and the ACEI/ARB group [RR = 1.93, 95% CI (1.02, 3.68), Z = 2.01, *P* = 0.04] (Figure 2D).

### Hypotension

The data of the PARAMOUNT study, the PARADIGM-HF study and the Parameter Study were collected and analyzed for the difference of hypotension between LCZ696 and ACEI/ARB. The heterogeneity test result of these studies was calculated as I^2^ = 57%. Therefore, the random effect model was used and the results demonstrated that there was still no difference between the LCZ696 group and the ACEI/ARB group [RR = 1.23, 95% CI (0.80, 1.89), Z = 0.96, *P* = 0.34] (Figure 2E).

### Hyperkalemia

Only the PARAMOUNT study, the PARADIGM-HF study and the Parameter Study reported the occurrence of hyperkalemia. The heterogeneity test result of these studies was calculated as I^2^ = 0%. Therefore, the fixed effect model was used for further analyses, and the results demonstrated that there was no difference between the LCZ696 group and the ACEI/ARB group [RR = 0.95, 95% CI (0.86, 1.04), Z = 1.17, *P* = 0.24] (Figure 2F).

### Renal dysfunction

Only the PARAMOUNT study and the PARADIGM-HF study reported the occurrence of renal dysfunction. The heterogeneity test result of these studies was calculated as I^2^ = 0%. The fixed effect model results demonstrated that the LCZ696 group significantly decreased renal dysfunction compared with the ACEI/ARB group [RR = 0.73, 95% CI (0.59, 0.91), Z = 2.87, *P* = 0.004] (Figure 2G).

### Other adverse effects

**Figure 3:**
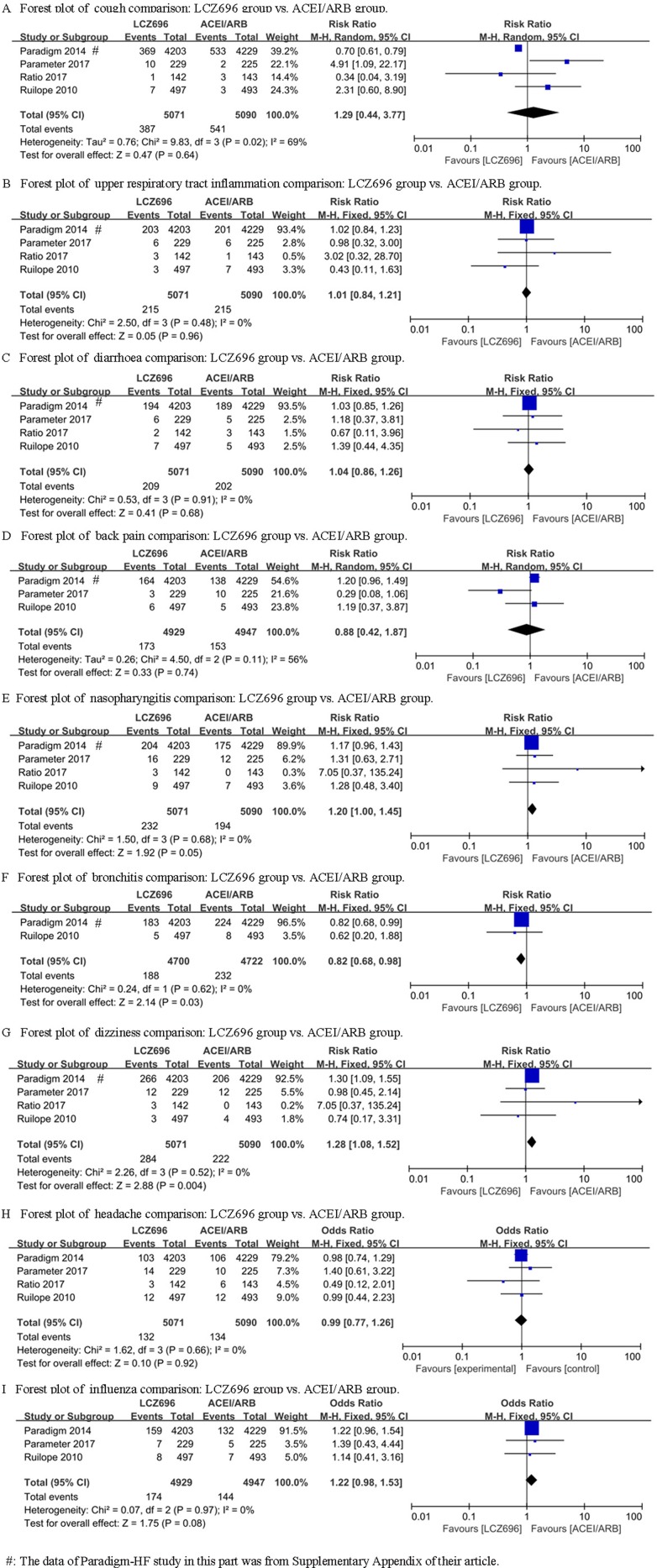
Forest plots depicting the comparison of LCZ696 and a placebo or ACEI/ARB on other adverse effects

The PARAMOUNT study didn’t explicitly provide the data involving other adverse effects, while the other 4 studies (Ruilope’s study, PARADIGM-HF study, Ratio study and Parameter Study) mentioned the adverse effects of cough, dizziness, upper respiratory tract inflammation, diarrhoea, bronchitis, back pain, nasopharyngitis, headache and influenza. For cough and back pain, random effect model were used (I^2^ = 69% and I^2^ = 56%) and the results showed no difference between the LCZ696 group and the ACEI/ARB group [RR = 1.29, 95% CI (0.44, 3.77), Z = 0.47, P = 0.64; RR = 0.88, 95% CI (0.42, 1.87), Z = 0.33, *P* = 0.74] (Figure 3A and 3D). For upper respiratory tract inflammation, diarrhoea, nasopharyngitis, headache and influenza, fixed effect model was used and the results also showed no difference between the LCZ696 group and the ACEI/ARB group [respectively: RR = 1.01, 95% CI (0.84, 1.21), Z = 0.05, *P* = 0.96; RR = 1.04, 95% CI (0.86, 1.26), Z = 0.41, *P* = 0.68; RR = 1.20, 95% CI (1.00, 1.45), Z = 1.92, *P* = 0.05; RR = 0.99, 95% CI (0.77, 1.26), Z = 0.10, *P* = 0.92; RR = 1.22, 95% CI (0.98, 1.53), Z =1.75, *P* = 0.08] (Figure 3B, 3C, 3E, 3H, 3I). LCZ696 significantly decreased the occurrence risk of bronchitis compared with ACEI/ARB [fixed effect model, respectively: RR = 0.82, 95% CI (0.68, 0.98), Z = 2.14, *P* = 0.03] (Figure 3F). However, LCZ696 significantly increased the occurrence risk of dizziness compared with ACEI/ARB [RR = 1.28, 95% CI (1.08, 1.52), Z = 2.88, *P* = 0.004] (Figure 3G).

## DISCUSSION

The main findings were as follows: (1) individuals assigned LCZ696 showed a statistically decreased risk of death compared with those assigned ACEI/ARB; (2) treatment with LCZ696 significantly decreased the risk of serious adverse events, discontinuation of treatment for any adverse event, renal dysfunction and bronchitis compared with a placebo or ACEI/ARB treatment; (3) LCZ696 significantly increased the risk of angioedema and dizziness; (4) for any adverse event, hypotension, hyperkalemia, cough, upper respiratory tract inflammation, diarrhoea, back pain, nasopharyngitis, headache and influenza, there was no significant difference between groups.

Recently, both ACC/AHA/HFSA [15] and ESC [16] guidelines for treatment of heart failure gave LCZ696 a class I level B recommendation based on the evidence of the PARADIGM-HF study. The guidelines also explicitly states that ARNI should not be administered concomitantly with ACEI or within 36 hours of the last dose of an ACEI, and should not be administered to patients with a history of angioedema, which mainly based on the trials of omapatrilat which was associated with a higher frequency and unacceptable incidence of angioedema [6]. In the PARADIGM-HF study, although they have excluded the participants who could not tolerate the therapy of LCZ696 or enalapril during an active run-in phase of 5–9 weeks, the incidence of angioedema was still seemed higher in the LCZ696 group than the enalapril group although without statistical significance. In the present study, we only wanted to know whether LCZ696, rather than ARNI, could increase the risk of angioedema, so we didn’t include the studies involving omapatrilat. The results suggested that LCZ696 statistically increased the risk of angioedema, but the *P* value was just 0.04. In the studies of Ruilope’s study and Kario’s study, no case of angioedema was found during the 8 weeks experimental period. The PARAMOUNT study reported only 1 case of angioedema in LCZ696 group during the 36 weeks and and Ratio Study also reported only 1 case of angioedema in valsartan group during the 8 weeks experimental period. Thus, the results of the present meta-analysis mainly from the PARADIGM-HF study and Parameter study. We thought that the reason why the cases reported in Ruilope’s study, Kario’s study, PARAMOUNT study and Ratio Study were much less than the other 2 studies was the shorter follow-up period, which might lead to an unreliable conclusion.

Despite the superiority of LCZ696 over enalapril, symptomatic hypotension has been seen as another important adverse effect that restricted the further application of LCZ696 in clinical practice. In the PARADIGM-HF study, symptomatic hypotension was more often present in the LCZ696 group compared with the enalapril group (14% vs 9.2%, *P* < 0.001). However, hypotension seems less occurred in LCZ696 group compared with olmesartan group in Parameter study (0.9% vs 2.2%), and there was no difference between groups in PARAMOUNT study (19% vs 18%, P=0.88). Integrating the above data, we obtained the result that the incidence of hypotension in the LCZ696 group was not higher than the ACEI/ARB group (*P* = 0.34). We thought about the results above, and speculated the reason of the difference might because of the difference of the enrolled population. The PARAMOUNT study enrolled heart failure with a preserved ejection fraction, and the PARADIGM-HF study enrolled heart failure with a reduced ejection fraction, while the Parameter study enrolled patients with only hypertension.

Although Fiona Bodey’s meta-analysis, which included 4 studies: the IMPRESS study [17], the OVERTURE study [18], the PARAMOUNT study and the PARADIGM-HF study, has demonstrated that ARNI decreased relative risk of renal dysfunction in heart failure compared to ACEI or ARB alone by 32%[19], the risk of renal insufficiency was still emphasized in the ACC/AHA/HFSA’s update [15]. The reason why LCZ696 was connected with renal impairment might be the greater hypotensive effect. However, clinical increases in the serum creatinine level and discontinuation for renal impairment were less [9], or at least no more [10], frequent in the LCZ696 group than in the ACEI/ARB group. In the present study, we only aimed to consider the effect of LCZ696 on renal dysfunction, so we extracted the relevant data of the PARAMOUNT study and the PARADIGM-HF study. The result again confirmed the significant protective effect of LCZ696 on renal function over ACEI or ARB alone. However, since the data we used were from the 2 above studies, it cannot be seen as a new discovery, and the conclusion still need to be confirmed by new investigations.

The most important finding in the present meta-analysis is the confirmation of LCZ696 on any adverse event, serious adverse event and discontinuation of treatment for any adverse event. Integrating all of the 6 studies, we found that no matter compared of a placebo or ACEI/ARB, LCZ696 didn’t increase the risk of any adverse events, which proved the safety of the drug. Simultaneously, to our surprise, fewer patients displayed serious adverse events, and fewer patients stopped their study medication for an adverse event in the LCZ696 group than in the ACEI/ARB group. Even compared with the placebo group, LCZ696 treatment also showed a smaller risk of discontinuation of the medication, which greatly encouraged us. In addition, we also summarized the results of other adverse effects repeatedly mentioned in the studies. Compared with the ACEI/ARB group, LCZ696 decreased the occurrence of the risk of bronchitis, but increased the occurrence of the risk of dizziness, which might need us to pay more attention. There was no difference for cough, upper respiratory tract inflammation, diarrhoea, back pain, nasopharyngitis, headache and influenza.

It is worth mentioning that Neprilysin is a major enzyme responsible for the degradation of Amyloid β (Aβ) peptide. Accumulation of toxic levels of Aβ in the brain leads to the dementia cases in the elderly population. In the previous study, although neprilysin knockout in mice has been confirmed to be responsible for the impairment of cognitive function [20], neprilysin overexpression did not improve deficits in spatial learning and memory in neprilysin transgenic mice [21]. The authors claimed that cognition, memory, and dementia-related adverse events were not increased in the LCZ696 group in the PARADIGM-HF study in their reply to reviewers [9]. And they thought that it was possible that cognitive decline related to vascular disease might be reduced by LCZ696. In addition, none of the 6 studies included in the present meta-analysis related the occurrence of the cognitive function impairment. Another large double-blind, parallel-group RCT, the PARAGON-HF study, which enrolled 4300 patients over a maximum follow-up period of 57 months, including repeated measurements of cognitive function in patients, might provide more evidences in cognitive function.

There are a number of limitations to our meta-analysis. First, in order to minimize the heterogeneity and bias, we used strict selection criteria, and thus only 6 randomized controlled trials of LCZ696 met the inclusion criteria. Furthermore, although the number of participants included in the study reached 11,821, most of them were from the PARDIGM-HF study. In addition, except for PARDIGM-HF study (27 months) and Parameter study (52 weeks), the other studies were generally short, so the results on the adverse effects only reflected the short-term effects of LCZ696. Therefore, we are anticipating the new larger trials of LCZ696, such as the PARAGON study and the UKHARP study, which will provide more useful information for us.

## MATERIALS AND METHODS

### Data source, search strategy, and inclusion criteria

**Figure 4:**
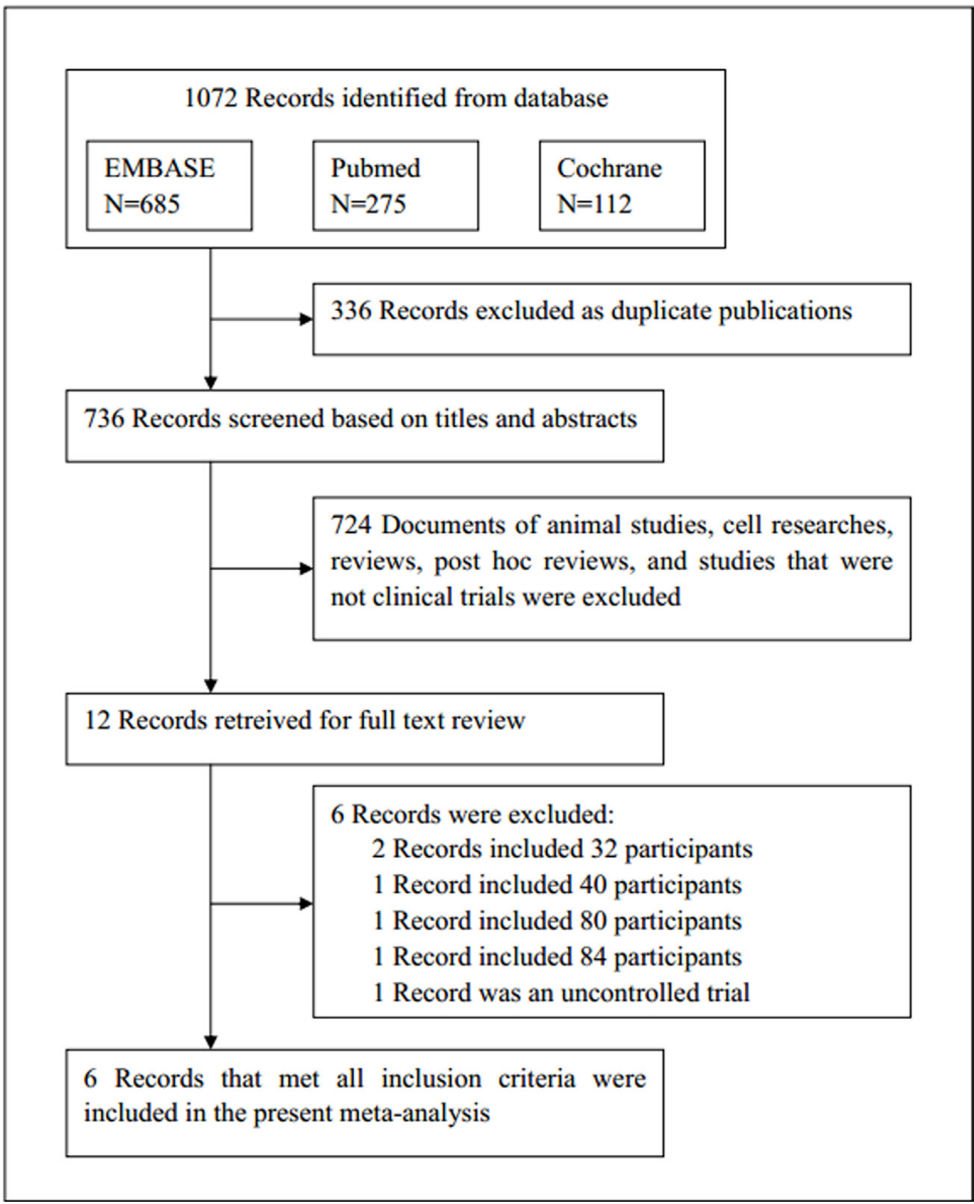
Flow diagram of the study selection process

**Figure 5:**
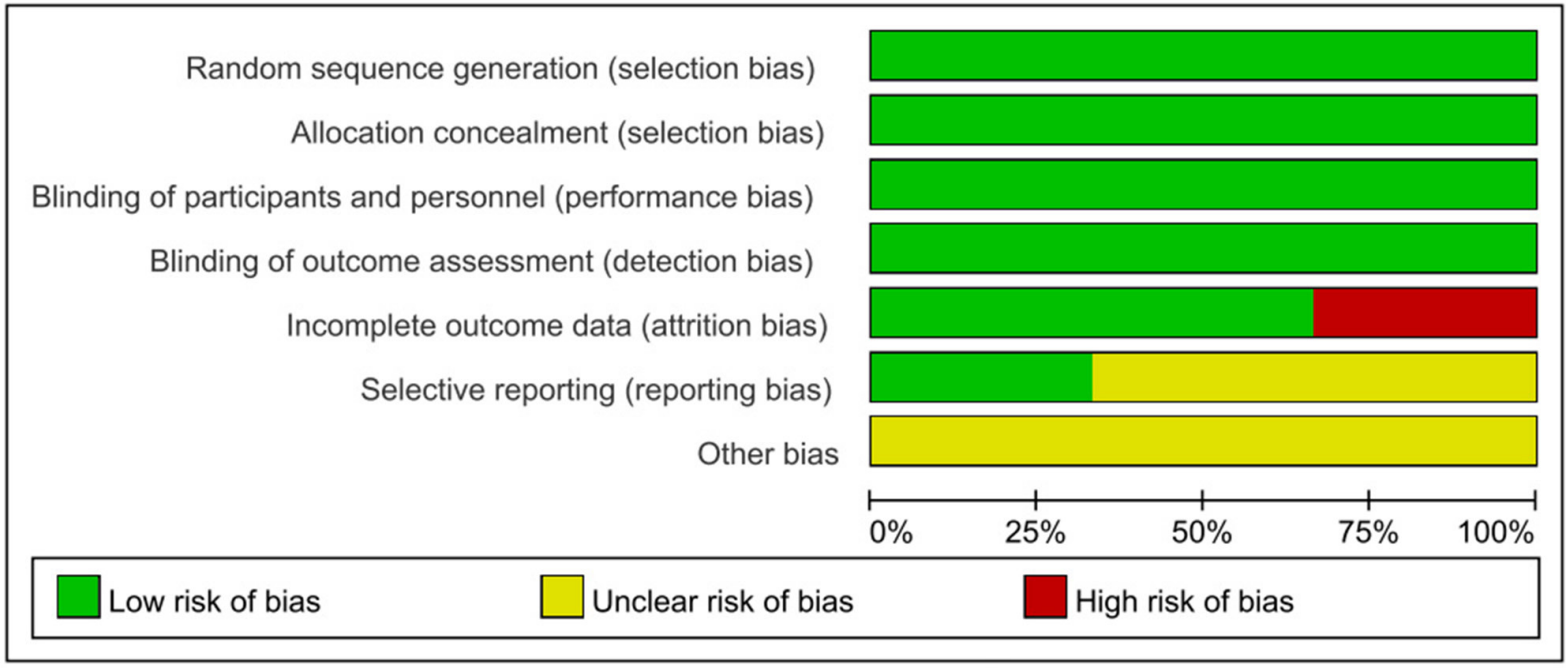
Risk of bias graph: review authors' judgements about each risk of bias item presented as percentages across all included studies

**Figure 6:**
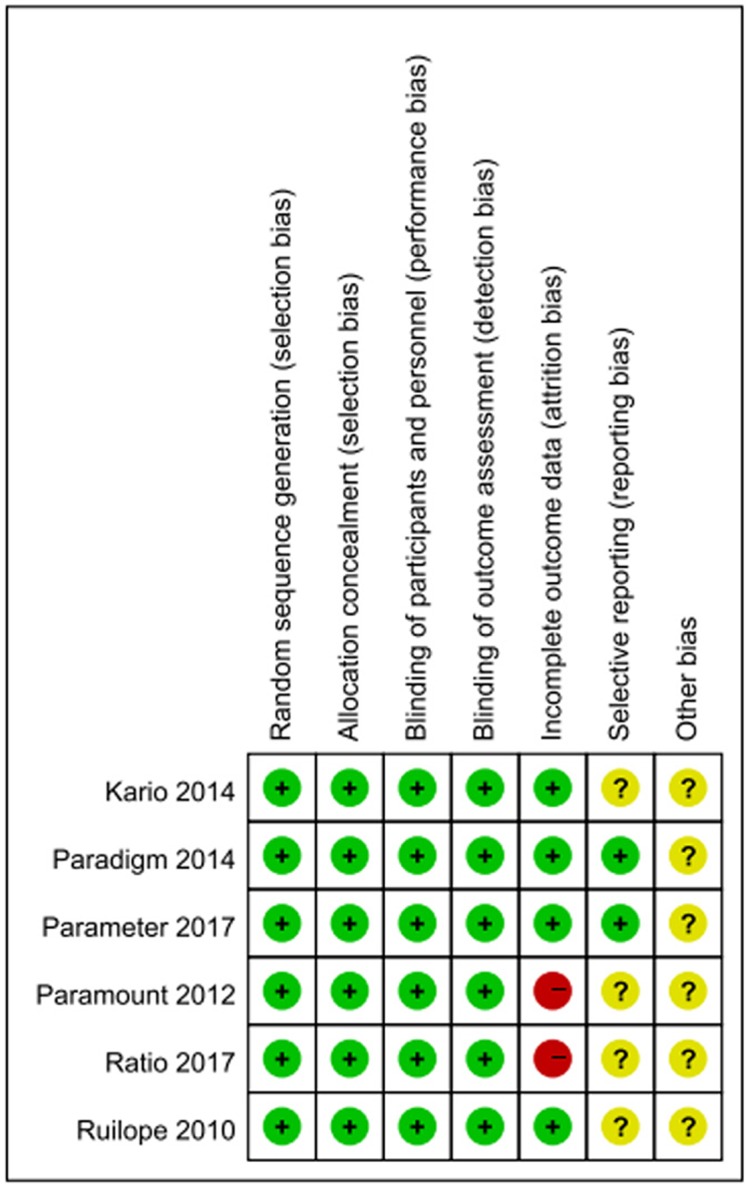
Risk of bias summary: review authors' judgements about each risk of bias item for each included study

EMBASE, PubMed and the Cochrane Library of Trials were carefully searched from April 2010 to May 2017 for the study. The following search terms or key words were used alone or in combination: ‘sacubitril/valsartan’, ‘LCZ696’, ‘neprilysin inhibitor’, ‘AHU377’, ‘valsartan’, ‘enalaprilat’, ‘hypertension’ and ‘heart failure’. After initially identifying 1072 potential trials, 336 duplicate documents were identified and 724 documents that were not clinical trials were excluded. The remaining 12 trials were carefully evaluated and 6 trials were excluded because there were less than 100 participants or they were non-controlled trials. Finally, a total of 6 eligible RCTs with 11,821 patients were included (Figure 4). All the 6 articles were published in English, conducted on human subjects, and classified as RCTs (Table 1). All of the 6 studies were at a lower risk of bias. These studies were conducted according to published protocols and randomization, double blinding, controlling, intention-to treat (ITT) were all performed for all of these studies. Risk of bias analysis was shown in Figures 5 and 6.

**Table 1:** Baseline characteristics of the 4 included trials

Study	Country	Population	LCZ696 Dose (mg/d)	Control group and Dose (mg/d)	Age	LCZ696 group (n)	Control group (n)	Follow up	End-point
Ruilope 2010	18 countries	Hypertension	100–400 mg qd	Valsartan 80–320mg qd/ AHU377 200mg qd/ placebo	18–75 y	497	493/165/172	8 w	Sitting diastolic blood pressure
Paramount 2012	13 countries	Heart Failure	200 mg bid	Valsartan 160 mg bid	≥ 40 y	149	152	36 w	Death
Kavio 2014	5 Asian countries	Hypertension	100–400 mg qd	placebo	≥ 18 y	297	92	8 w	Diastolic BP
Paradigm 2014	47 countries	Heart Failure	200 mg bid	Enalapril 10 mg bid	18–96 y	4187	4212	27 m	Death
Ratio 2017	9 countries	Hypertension	400 mg qd	Valsartan 320 mg qd	≥ 18 y	142	143	8 w	Sitting systolic blood pressure
Parameter 2017	12 countries	Hypertension	400 mg qd	Olmesartan 40mg qd	≥ 60 y	229	225	52w	central aortic systolic pressure

### Data synthesis and statistical analysis

All analyses were performed using RevMan software version 5.3. Pooled risk ratios (RR) with 95% CIs were presented for dichotomous outcomes (e.g. AEs, serious AEs, and discontinuation of participants in the trials, as well as hypotension, renal impairment and hyperkalemia). The results of the included studies were performed with fixed-effect models (Mantel–Haenszel method) [22] or random-effect models in cases of significant heterogeneity between estimates [23]. We used the I^2^ statistics to assess the magnitude of heterogeneity: 25%, 50%, and 75% represented low, moderate, and high degrees of heterogeneity respectively. The effect model chosen was based on the analysis results: the fixed effect model was used if I^2^ < 50% and the random effect model was used if I^2^ ≥ 50% [24].
